# Commentary: Description of Clinical Characteristics of VAP Patients in MIMIC Database

**DOI:** 10.3389/fphar.2021.736447

**Published:** 2021-09-09

**Authors:** Yun Li, Xuan Zhang

**Affiliations:** ^1^ Medical School of Chinese PLA, Beijing, China; ^2^ Department of Critical Care Medicine, The First Medical Centre, Chinese PLA General Hospital, Beijing, China; ^3^ Faculty of Hepato-Pancreato-Biliary Surgery, Chinese PLA General Hospital, Beijing, China

**Keywords:** ventilator-associated pneumonia, pathogenic bacteria, MIMIC database, colonization, infection

## Introduction

Ventilator-associated pneumonia (VAP) is a well-known complication of invasive mechanical ventilation in critically ill patients (Metersky and Kalil, 2018). We read with great interest the article entitled “*Description of Clinical Characteristics of VAP Patients in MIMIC Database* (Liu et al., 2019).” We would like to congratulate the authors for their application of the Medical Information Mart for Intensive Care (MIMIC) database to identify the clinical characteristics of VAP patients in ICU. However, we would like to make a few comments on the methodology and the interpretation of the findings of this study.

## Definition of Pathogen

In the abstract, the authors claimed that “The main pathogens were YEAST (16.71%), STAPH AUREUS COAG+ (11.63%), *Staphylococcus*, COAGULASE NEGATIVE (8.68%), GRAM NEGATIVE ROD (S) (6.14%), and *Pseudomonas aeruginosa* (5.73%).” We understood that this may just be the results they got from the database, but it should be interpreted properly.

For one thing, according to the traditional clinical microbiology, the description “STAPH AUREUS COAG+ (11.63%), *Staphylococcus*, COAGULASE NEGATIVE (8.68%)” is not appropriate, as the coagulase-positive *Staphylococci* includes *Staphylococcus aureus* and *Staphylococcus* lugdunensis. “GRAM NEGATIVE ROD (S) (6.14%)” using Gram negative *bacillus* might be more appropriate (Busse et al., 1996).

For another, in the MIMIC database, the sites of the infection/culture were specified. But in this report, it seems that the author did not include any of those data in the analysis. If the intention were to describe the distribution of pathogens in VAP, it may be useful to include this information. Because culture-positive could be colonizing microorganisms, which is largely site-specific (Jarvis, 1996).

Last but not least, the definition of the pathogen is “a specific causative agent of disease.” Culture positive means the presence of the microorganisms but they are not necessarily the causes of pneumonia or VAP (Messika et al., 2018). In this report focusing on VAP, however, yeast and coagulase-negative Staphylococci, etc., are mostly colonized on the respiratory system and rarely the cause of the infection (Guidelines for the manage, 2005). We understand these culture results were recorded in the database, but some interpretations are needed if those were supposed to be of guidance for diagnosis and treatment of VAP (Papazian et al., 2020).

## Methodology and Interpretation of Results

As for the methodology, we are not sure what was the purpose of categorizing patients based on gender. In Table 1, the authors compared the basic characters of study participants while summarized the basic characteristics in two columns. And we feel this is hardly justifiable. Is gender a risk factor or confounder for VAP according to previous studies? Furthermore, the author claimed, “Both males and females had a highest proportion of survival.” We are not sure what this indicates or to whom the highest “proportion of survival” was compared?

In the third paragraph of the result, “the rest were other pathogenic bacteria (52.83%).” In MIMIC, the specific types of pathogens were clearly recorded. As the authors listed yeast as the most common bacteria, we feel it could be likely that some of the important pathogens were inappropriately combined in this category.

In the fourth paragraph of the result, the authors listed “synthetic antibacterials” as a category of antibiotics. We feel this might not be appropriate. Several types of antibiotics were synthetic, covering different mechanisms (Mohr, 2016; Balaban et al., 2019). Classification based on whether the antibiotics were synthetic may be of limited clinical reference.

We understood that the study would be limited by the nature of MIMIC, a retrospective database. Again, we would like to congratulate the authors for a nicely done study.
